# Supplementary material for: Dietary inulin alters the gut microbiome, enhances systemic metabolism and reduces neuroinflammation in an APOE4 mouse model
Source: PLoS One. 2019 Aug 28;14(8):e0221828. doi: 10.1371/journal.pone.0221828 (PMC6713395; doi:10.1371/journal.pone.0221828)
Supplement: Attachment — Submitted filename: Inulin Rebuttal Letter_08.06.19_final_rev.docx [file pone.0221828.s001.docx]

**We thank the reviewers for their thoughtful comments. Below are the point-by-point responses and the changes are highlighted in yellow in the manuscript.**

Reviewer #1:

This is a very interesting paper showing how preserving metabolic function through microbiota modulation AD risk can be reduced. In my opinion the author should mention recent papers demonstrating that upon probiotics administration AD progression is counteracted based on microbiota modulation, reduction of inflammatory cytokinesm increased SCFAs, increased neuroprotective gut hormones (Bonfili L. et al. Scientific Reports 2017)

**Response: We thank the reviewer for the kind appraisal and for presenting this paper for our consideration. We have added the paper in the Introduction as an important reference (reference 16) and the above information in the Discussion (Lines 590-592).**

Reviewer #2:

1 The expression of APOE4 should be included after the adminstration of inulin. And more AD biomaker as Tau, BACE, p-Tau, or histopathology of brain and colon.

**Response: We appreciate the reviewer’s thoughtful comment. We acknowledge a limitation of the study is that we did not determine the APOE4 protein level after the Inulin diet intervention. We have added this in the Discussion as a future direction (Lines 597-602). Further, the EFAD mice used in the study do not express Tau (they only had human APOE and amyloid beta expression), therefore, we were not able to determine the Tau level in the present study. This is a limitation of the animal model. We have added this into Discussion as a potential future direction as well (Lines 597-602).**

2 I think there are at lest two does of inulin treated groups should be proved.

**Response: We thank the reviewer for the comment. We chose the 8% inulin level based on a dose-dependent study (4% vs. 8% inulin), which showed that 8% had higher beneficial effects than 4% (Zdunczyk et al., 2004; reference 28). We apologize that we did not include the details in the previous submission. The information has been added in this revision (Lines 104-108). On the other hand, the goal of the study was to determine whether a single dose of Inulin is sufficient to alter gut microbiome and systemic metabolism in the E4FAD mice before they developed cognitive impairment as there is not much information regarding Inulin’s effect on APOE4 carriers. However, we do appreciate the reviewer’s comment, and will be designing studies to investigate the dose-dependent metabolic changes in response to inulin in the future. We also add this as a future direction in Discussion (Line 602).**

3 What the dose judgment, 8% of inulin? AS: Vishal Singh et al., (2018), Dysregulated Microbial Fermentation of Soluble Fiber Induces Cholestatic Liver Cancer, Cell, DOI: <https://doi.org/10.1016/j.cell.2018.09.004>.

**Response: We thank the reviewer for the inquiry and paper. As mentioned above, we used 8% inulin based on the study by Zdunczyk et al. (reference 28). The information has been added in this revision (Lines 104-108).**

**We suspect that the 7.5% inulin had adverse effect on liver cancer shown in the Cell paper might due to the different animal model used and different disease investigated. We did not see adverse effects of 8% inulin in our study, potentially because our mice were still relatively healthy. Nonetheless, we thank the reviewer for pointing this paper to us.**

4 More the correction analysis bteween the AD biomaker and gut microbiota, Metabolite.

**Response: We appreciate the comment. As the study was focused on restoring metabolism at pre-clinical stage, the mice had not produced many AD biomarkers, such as Aβ. We addressed in the Dissuasion that *“In the future, it will be interesting to know if inulin is also able to halt the progression of Aβ after AD has developed. It will also be interesting to identify if inulin is effective in counteracting other AD pathologies, such as tau tangles, with other animal models.”* (Lines 598-601). At that stage, it will be definitely important to identify the correction analysis between AD biomarker and gut microbiota and metabolites.**

5 Are the metabolite of Scyllo-inositol and myo-inositol is related to AD?

**Response: Yes, scyllo-inositol is associated with Aβ inhibition and has been used in clinical trial to reduce Aβ aggregation. Significant increases in myo-inositol are seen in brains of AD patients. We have clarified this in the Results (Lines 462-464) and Discussion (Lines 557-560).**

Reviewer #3:

1. The references Nuzzo D, et al. Nutrients 2018 and Amato A et al. Nutrients 2017 should be added in the introduction to describe the link between oxidative stress and diseases.

**Response: We thank the reviewer for the thoughtful suggestion. We have added these references to the Introduction (references 14 and 15) and include the information in the Discussion (Lines 510-513).**

2. I suggest to give more information regarding the mouse treatment. The Authors may add some additional information like food consumption (g for a day) and total caloric intake. How many grams of inulin are daily supplied to mice?

**Response: We thank the reviewer for the comment. In Figure 1A, we demonstrated the food intake in kcal/day for each group and energy intake information was shown in the Table 1. The inulin fiber is contained in the chow diet and the mice had ad libtum access to the diet. The remaining diet was measured biweekly to estimate the food intake of the mice. We have added more information and clarified the issue in Methods (Lines 114-116).**

3. In Figure 1(D) the image of E3-control is missing.

**Response: We thank the reviewer for spotting this mistake. We have corrected the image.**

4. The title of the article is inappropriate because the data reported are insufficient to claim a potential role of inulin for the Alzheimer disease. I suggest to remove "[…] relevant mouse model of preclinical Alzheimer's Disease" from the title.

**Response: Thank you for this suggestion. We agree and have changed the title accordingly.**
